# A novel mutation in the *BCHE* gene and phenotype identified in a child with low butyrylcholinesterase activity: a case report

**DOI:** 10.1186/s12881-018-0561-5

**Published:** 2018-04-10

**Authors:** Rentao Yu, Yanzhi Guo, Yunjie Dan, Wenting Tan, Qing Mao, Guohong Deng

**Affiliations:** 1 Department of Infectious Diseases, Southwest Hospital, Army Medical University (Third Military Medical University), Chongqing, 400038 People’s Republic of China; 2 Chongqing Key Laboratory of Infectious Diseases, Southwest Hospital, Army Medical University (Third Military Medical University), Chongqing, 400038 China

**Keywords:** BCHE gene, Inherited butyrylcholinesterase deficiency, Genetic variant, Intellectual disability

## Abstract

**Background:**

Butyrylcholinesterase (BChE), an ester hydrolase produced mainly by the liver, hydrolyzes certain short-acting neuromuscular blocking agents, like succinylcholine and mivacurium that are widely used during anesthesia. Patients with BChE deficiency are possibly in danger of postanesthetic apnea. Hereditary BChE deficiency results from the mutations of *BCHE* gene located on chromosome 3, 3q26.1-q26.2, between nucleotides 165,490,692–165,555,260.

**Case presentation:**

This study describes a novel mutation in a child with BChE deficiency. In general, this child appeared healthy and well-developed with a normal appearance. However, the results of Wechsler Intelligence Scale showed that the full-scale intelligence quotient (FIQ) was 53, classified into the group with the minor defect. The BChE activity was 32.0 U/L, considerably lower than the normal lower limit (reference range: 5000-12,000 U/L). Sanger sequencing showed that there were 2 mutations in the exon 2 of *BCHE* gene of this child. One is a heterozygous mutation rs764588882 (NM_000055.3: c.401_402insA, p.Asn134Lysfs*23). The other one is a heterozygous mutation (NM_000055.3: c.73A > T, p.Lys25Ter) that has never been reported before. The two mutations lead to a premature stop of transcription.

**Conclusions:**

Double heterozygous recessive mutations are the cause of BChE deficiency of this boy in this study, including a novel mutation c.73A > T. Intellectual disability is a new phenotype that is probably associated with this mutation.

## Background

Acetylcholinesterase (AChE) and butyrylcholinesterase (BChE) are the two types of cholinesterases in humans [1]. Unlike AChE, BChE is an ester hydrolase produced mainly by the liver and then distributes to all tissues. Functionally, BChE hydrolyzes certain short-acting neuromuscular blocking agents, succinylcholine and mivacurium widely used during anesthesia, as well as some drugs like procaine, tetracaine and heroin [2]. Interestingly, patients with BChE deficiency have no life risks and are healthy in normal conditions. However, they are likely to be in danger of postanesthetic apnea, presenting as prolonged coma even death after the administration of succinylcholine or mivacurium [3, 4]. There are several factors that cause low BChE activity. Except for external factors, hereditary BChE deficiency may also lead to a low BChE activity in plasma.

In this paper, we describe a child with BChE deficiency identified by health examinations. Sanger sequencing identified a novel mutation that has never been reported. Besides, a novel phenotype presented by this child has not been described, either. We hope this report could provide new evidence and clues to the genetic variants and phenotype of BChE deficiency. This study was approved by the ethics committee of Southwest Hospital, Chongqing, China. Informed consents were obtained from his parents. The study protocol conforms to the ethical guidelines of the 1975 Declaration of Helsinki.

## Case presentation

This case was drafted and outlined in accordance with the CARE guidelines, which regulate the discipline of case reports. A 14-year-old boy, in his junior high school, visited the local hospital for routine physical examination. After regular biochemical examinations, we noticed an extremely low level of BChE activity for his liver function tests. Except for his parents, this child has a younger brother in the family. His four grandparents are still alive, but living in the remote district and did not come for tests. In general, this child appeared healthy and well-developed with a normal appearance. He had a soft abdomen and normal-sized liver with no tenderness and rebound tenderness. He had no other signs of liver injury or chronic liver disease. Based on his performance, Wechsler Intelligence Scale for Children (Chinese edition) was used for this child by qualified psychological doctor [5, 6]. The results showed that verbal intelligence quotient (VIQ) of this child was 51, and performance intelligence quotient (PIQ) was 57, and the full-scale intelligence quotient (FIQ) was 53. Finally, he was classified into the group with the minor defect based on the result. This is the first case report about the association between BCHE deficiency and intellectual disability. The results of the remaining examination were normal.

Biochemical examinations revealed that this child had a normal blood level of aspartate aminotransferase (AST, 24.4 IU/L, reference range: 0-42 IU/L) and alanine aminotransferase (ALT, 27.4 IU/L, reference range: 0-42 IU/L) but an elevated blood level of alkaline phosphatase (ALP, 279 IU/L, reference range: 34-114 IU/L). The BChE activity was measured by means of colorimetric butyrylthiocholine with butyrylcholinesterase kit [7] at Hitachi 7600 chemistry auto-analyzer. Each step was conducted according to manufacturer’s instructions. The BChE activity was 32.0 U/L, considerably lower than the normal lower limit (reference range: 5000-12,000 U/L). Tests for hepatitis A virus (HAV) IgM antibodies, hepatitis B virus surface antigen (HBsAg), hepatitis B virus core antibodies (HBcAb), hepatitis C virus (HCV) antibodies were negative. The hepatitis B virus surface antibodies (HBsAb) were positive. Other laboratory examination results are shown in Table 1. Ultrasonography of the abdomen showed a normal result of each organ. The other members of his family had no critical abnormality and laboratory tests revealed normal results. The levels of BChE activity of each member are listed in Table 2.

**Table 1:** Laboratory data on presentation

Variable	On presentation	Reference range
Hematocrit (%)	38.0	35-55
Hemoglobin (g/L)	132	110-160
White-cell count (10^9^/L)	4.25	4-10
Differential count (%)		
Neutrophils	33.2	50-70
Lymphocytes	64.5	20-40
Monocytes	1.4	3-8
Eosinophils	0.9	0.5-5
Basophils	0	0-1
Platelet count (10^9^/L)	128	100-300
Red blood cell count (10^12^/L)	4.27	3.5-5.5
Reticulocytes (%)	1.50	0.5-1.5
Mean corpuscular volume (fL)	88.3	75-110
Mean corpuscular hemoglobin (pg)	30.9	26-38
Mean corpuscular hemoglobin concentration (g/L)	350	310-370
Red blood cell distribution width (%)	13.0	11.6-14.6
Calcium (mmol/L)	2.59	2.1-2.6
Phosphorus (mmol/L)	1.71	0.81-1.55
Total protein (g/L)	72.15	66-83
Albumin (g/L)	44.54	38-51
Globulin (g/L)	27.61	25-38
Alanine aminotransferase (IU/L)	27.4	0-42
Aspartate aminotransferase (IU/L)	24.4	0-42
Alkaline phosphatase (IU/L)	279	34-114
Total bilirubin (umol/L)	17.92	6-21
Direct bilirubin (umol/L)	4.09	0-6
Indirect bilirubin (umol/L)	13.83	3-16
γ-Glutamyltransferase (IU/L)	29	4-50
Lactate dehydrogenase (IU/L)	195.9	114-240

**Table 2:** BChE activity and genetic variants of *BCHE* gene identified by Sanger sequencing

Subjects	BChE activity(U/L)	Exon 1	Exon 2	Exon 3	`Exon 4
Father	8606.0	None	c.73A > T A/T, p.Lys25Ter, no report	None	1) c.1699G > A G/A, p.Ala567Thr, rs1803274,1000G frequency:CHB(A = 16.99%)/CHS(A = 10.95%)* 2) 3’UTR *189G > A G/A, rs3495, 1000G frequency:CHB(A = 76.21%)/CHS(A = 78.57%) 3) 3’UTR *288_289insT T/T, rs397762799
Mother	5003.0	None	c.401_402insA A/−, p.Asn134Lysfs*23, rs764588882	None	1) 3’UTR *189G > A A/A, rs3495, 1000G frequency:CHB(A = 76.21%)/CHS(A = 78.57%) 2) 3’UTR *288_289insT T/T, rs397762799
Brother	11,322.0	None	None	None	1) c.1699G > A G/A, p.Ala567Thr, rs1803274, 1000G frequency:CHB(A = 16.99%)/CHS(A = 10.95%) 2) 3’UTR *189G > A G/A, rs3495 1000G frequency:CHB(A = 76.21%)/CHS(A = 78.57%) 3) 3’UTR *288_289insT T/T, rs397762799
Patient	32.0	None	1) c.73A > T A/T, p.Lys25Ter 2) c.401_402insA A/−, p.Asn134Lysfs*23, rs764588882	None	1) 3’UTR *189G > A G/A, rs3495, 1000G frequency:CHB(A = 76.21%)/CHS(A = 78.57%) 2) 3’UTR *288_289insT T/T, rs397762799

*Population data were cited from the dbSNP database of NCBI

**Fig. 1:**
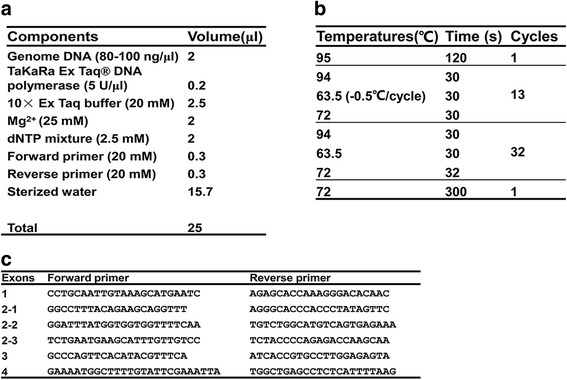
The reagents, conditions and primers of PCR. **a** The components and volumes of PCR for *BCHE* gene. **b** The conditions of touchdown PCR. **c** The primer sequences of each exon of *BCHE* gene

**Fig. 2:**
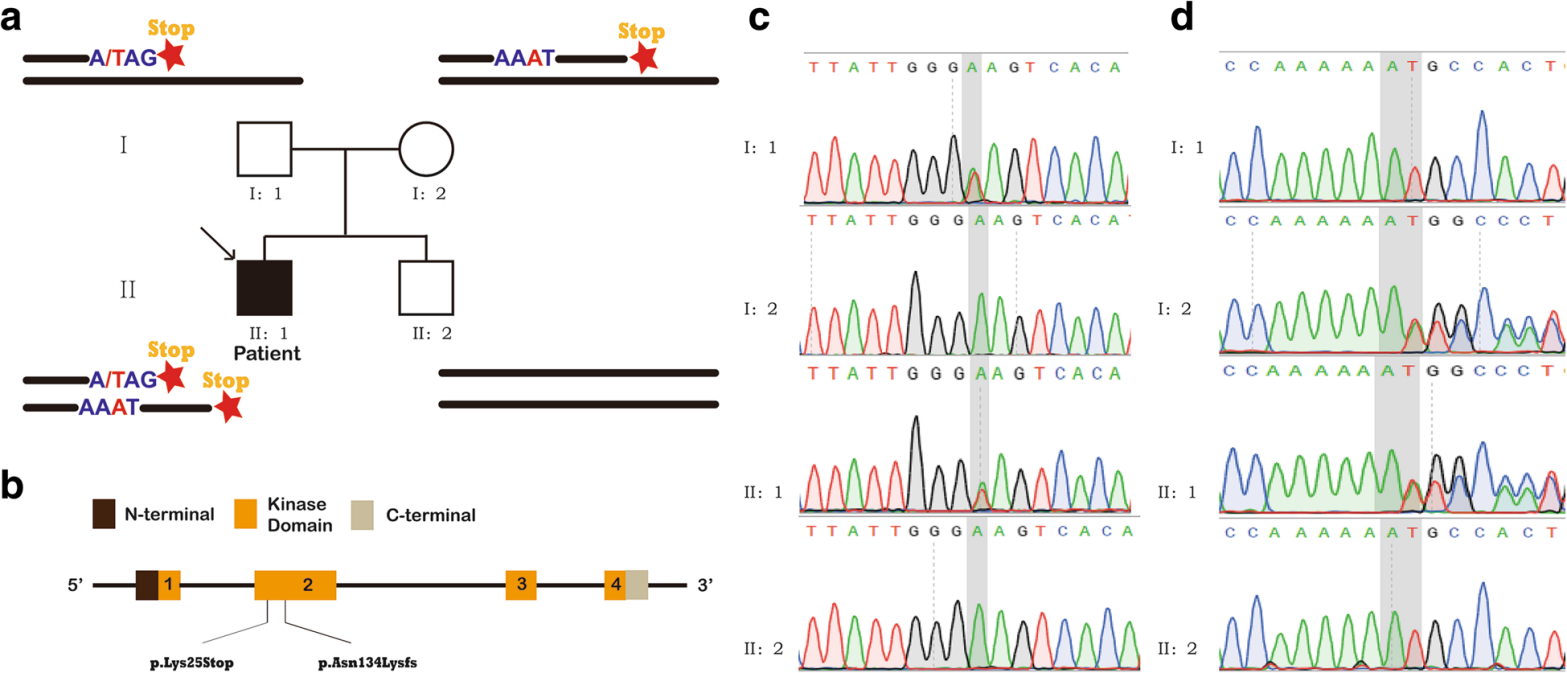
Genogram, mutation patterns and electropherograms of the index patient. **a** The genogram showed an isolated patient. Roman numerals indicate generations and Arabic numbers indicate individuals. Squares = males, circles = females. Affected individuals are denoted by solid symbols and unaffected individuals are denoted by open symbols. The index patient is indicated by an arrow. The two mutations were inherited from father and mother respectively. **b** The structure of *BCHE* gene. This novel mutation is within exon 2. **c** The electropherograms of the mutation c.73A > T of exon 2. The shadowed column was mutation point. **d** The electropherograms of the mutation c.401_402insA of exon 2. The shadowed column was insertion point

To fully understand this abnormality, Sanger sequencing was applied to identifying potential variants that may affect BChE activity. He, his parents and his brother were withdrawn blood and then DNA was extracted for Sanger sequencing. Finally, the exonic regions of *BCHE* gene linked to BChE deficiency was chosen for sequencing in this family. The platform for Sanger sequencing is Applied Biosystems® 3730 DNA Analyzer by using BigDye™ Terminator v3.1 Cycle Sequencing Kit. *BCHE* gene contains 4 exons and 6 pairs of primers were designed and amplified at these four exons (Exon 2 is long and 3 pairs of primers were designed). The specific primer sequences and details of PCR are listed in Fig. 1. Table 2 shows the genetic variants identified by Sanger sequencing. There are 2 mutations in the exon 2 of BCHE gene of this child. One is a heterozygous mutation rs764588882 (NM_000055.3: c.401_402insA, p.Asn134Lysfs*23). The insertion of a base A introduces a frameshift mutation in the open reading frame, which results a stop Codon at the 23rd amino acids downstream of the insertion point. The other variant is a novel heterozygous mutation (NM_000055.3: c.73A > T, p.Lys25Ter), which introduces a stop Codon at the same locus (stop at the 25th amino acid lysine). These two heterozygous mutations were also identified in his mother (c.401_402insA, p.Asn134Lysfs*23, heterozygous) and father (c.73A > T, p.Lys25Ter, heterozygous) respectively, suggesting that the two mutations distribute in the two chromosomes respectively. The exon 2 of *BCHE* gene in this child’s younger brother had no mutations in both of the two chromosomes. Combined with the serum BChE activity of his brother, we could reach to the hereditary pattern of this disorder in the family. As for exon 4, there are three single nucleotide polymorphisms (SNP) identified in this family, rs1803274 (NM_000055.3: c.1699G > A, p.Ala567Thr), rs3495 (NM_000055.3: 3’UTR *189G > A) and rs397762799 (NM_000055.3: 3’UTR *288_289insT), all of which are benign mutations as indicated by ClinVar database from National Center for Biotechnology Information (NCBI). Above all, it was the mutations in exon 2 but not exon 4 that caused BChE deficiency of this child. The electropherograms and sequencing results with comparison and hereditary pattern are shown in Fig. 2. Finally, this boy was diagnosed with hereditary BChE deficiency. Since this is a benign disease and has no significant influence on the health of this boy, no specific medical interventions were applied to him, but he and his parents were told the risk of prolonged apnea after anesthesia.

## Discussion and Conclusions

In summary, there are three types of causes that lead to reduced activity of BChE. The major cause is postnatal factors, especially the factors that induce liver injury. The most common reasons include liver and kidney disease, dystrophy, burn, malignancy, hypothermia, uremia and pregnancy [8–11]. The secondly dominant reason is drug-related factors. Some drugs have been reported to cause BChE deficiency and anticholinesterase drugs are the major types. Other drugs include contraceptive agents, glucocorticoid, monoamine oxidase inhibitor, esmolol, etc. [12] The remainder is genetic factors. This child had no signs of liver injury or severe diseases and he had never been administered these drugs, either. Consequently, we assume that this case belongs to the third category. Hereditary BChE deficiency results from the mutations of *BCHE* gene located on chromosome 3, 3q26.1-q26.2, between nucleotides 165,490,692–165,555,260 [13]. Over 20 genetic variants in *BCHE* gene have been identified to link with this type of disease, including qualitative variants with altered hydrolyzing activity, the atypical (A) [14], fluoride-resistant (F) [15], and silent (S) variants [16], etc. and quantitative variants with decreased enzyme concentration but normal activity, like H [17], J [18], and K variants [19].

In this study, we reported a new mutation that led to BChE deficiency. Despite that the mutations in *BCHE* gene are recessive, the double heterozygous recessive mutations of Exon 2, c.73A > T and c.401_402insA, occurred in the two chromosomes respectively caused the inactivity of BChE of this boy. Both mutations caused a precurrent stop of transcription, leading to a premature or inactive protein/ polypeptide.

Intellectual disability is the phenotype that was first found associated with BChE deficiency in our study. Although the exact mechanism still remains unknown, there did have been some studies implicating the possible connection. Animal experiments suggested that BChE together with AChE participates in the regulation of dorsal root ganglia, the growth of axons, nerve terminals and cones and other processes of neurite outgrowth [20]. Besides, BChE is reported to play a role in dementia and Alzheimer’s Disease (AD). In the mild AD, lower functional BChE levels in cerebrospinal fluid are linked to high Aβ-brain load and impaired cognitive performance. Especially, the K variants of BChE, which hinder the ability to interact with Aβ, is predominantly associated with the progression of AD [21]. Based on these previous studies, we assumed that there is an association between this new variant and the phenotype of intellectual disability. However, further studies should be conducted to confirm this connection.

Conclusively, double heterozygous recessive mutations are the cause of BChE deficiency of this boy in this study, including a novel mutation c.73A > T. Generally, BChE deficiency is a benign disease, however, patients with BChE deficiency would be in danger of prolonged coma and respiratory depression. Besides, intellectual disability is another phenotype that probably should be considered and may be associated with this mutation. Based on this reason, it is important to determine the gene variants and heredity patterns. We hope that our findings could add new clues to this disease.

## Abbreviations

AChE: Acetylcholinesterase
BChE: Butyrylcholinesterase
HBV: Hepatitis B virus
IQ: Intelligence quotient
SNP: Single nucleotide polymorphisms
